# Supplementary material for: Fn-OMV potentiates ZBP1-mediated PANoptosis triggered by oncolytic HSV-1 to fuel antitumor immunity
Source: Nat Commun. 2024 Apr 30;15:3669. doi: 10.1038/s41467-024-48032-7 (PMC11063137; doi:10.1038/s41467-024-48032-7)
Supplement: Supplementary file 1 — Supplemental Information [file 41467_2024_48032_MOESM1_ESM.pdf]

Supplementary Information for "*Fn*-OMV potentiates ZBP1-mediated PANoptosis triggered by oncolytic HSV-1 to fuel antitumor immunity"

Shuo Wang [1,#], An Song [1,#], Jun Xie [1,2], Yuan-Yuan Wang [1], Wen-Da Wang [1], Meng-Jie Zhang [1], Zhi-Zhong Wu [1], Qi-Chao Yang [1], Hao Li[1], Junjie Zhang[1,2,*], Zhi-Jun Sun[1,*]

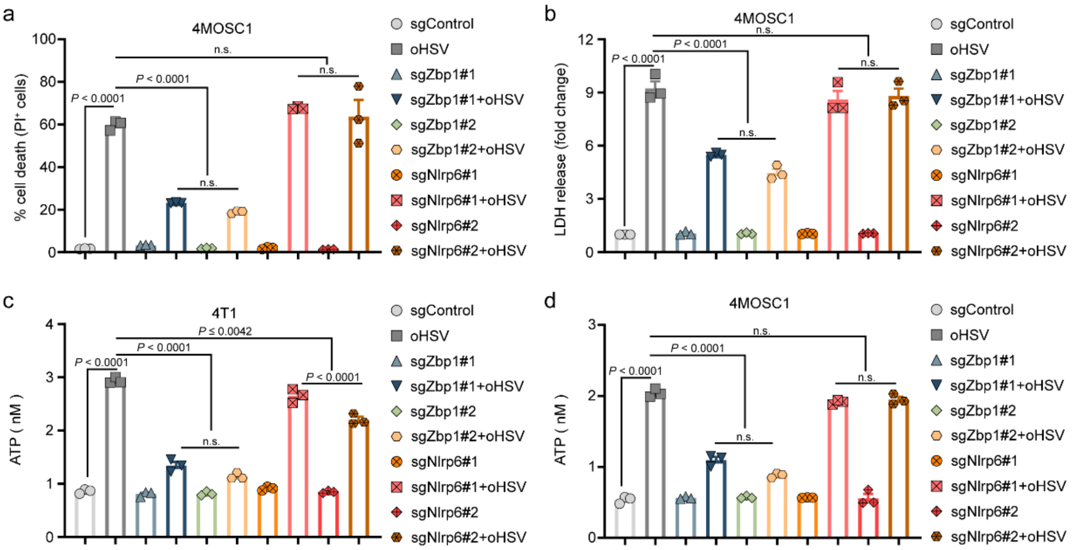

Supplementary Fig 1. oHSV induces ZBP1-mediated tumor cell death. a The effect of ZBP1 and NLRP6 expression on relative cell viability in the 4MOSC1 cells treated with oHSV (MOI = 1) for 24 h. **b** The effect of ZBP1 and NLRP6 expression on the injury of 4T1 cells after oHSV (MOI = 1) treatment for 24 h was detected by LDH release assay. **c** The effect of ZBP1 and NLRP6 expression on ATP released by 4T1 cells treated or untreated with oHSV (MOI = 1) for 24 h. **d** The effect of ZBP1 and NLRP6 expression on ATP released by 4MOSC1 cells treated or untreated with oHSV (MOI = 1) for 24 h. All data are shown as the mean ± s.e.m. n = 3 independent experiments for **a-d**. Statistical significance was calculated via one-way ANOVA with Tukey's multiple comparisons test. n.s. not significant. Source data are provided as a Source Data file.

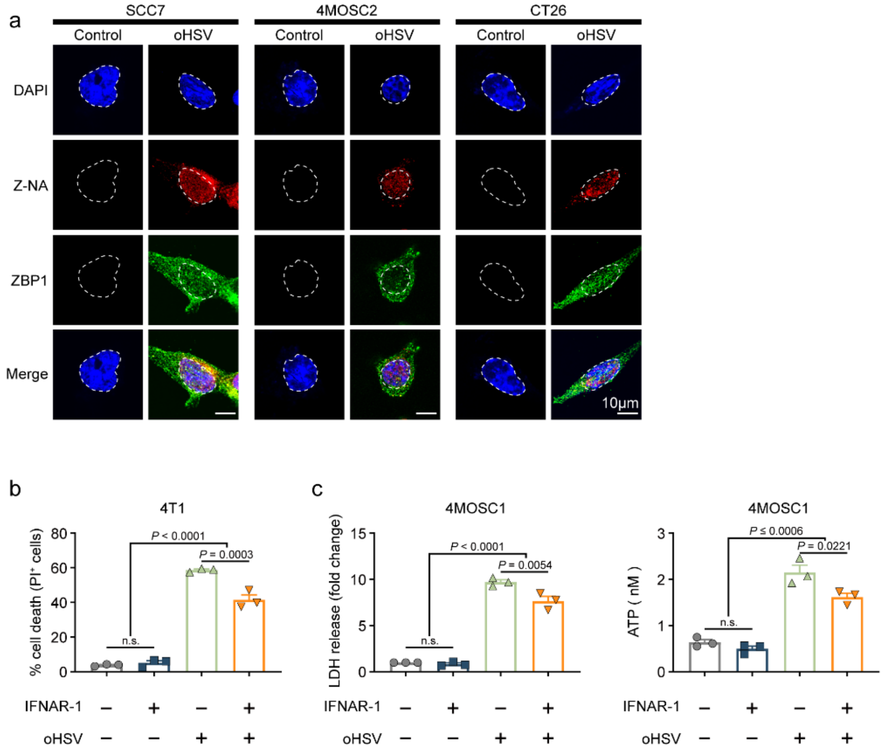

Supplementary Fig 2. The expression of Z-NA and ZBP1 in tumor cells after oHSV treatment. a The expression of Z-NA and ZBP1 in SCC7, 4MOSC2 and CT26 tumor cells after oHSV (MOI = 1, 24 h) treatment was evaluated using a Confocal Laser Scanning Microscope. Scale bars = 10 μm. Data were repeated thrice independently with similar results. **b** The effect of IFNAR-1 on relative cell viability in the 4T1 cells treated with oHSV (MOI = 1) for 24 h. The release of LDH (left) and ATP (right) from 4MOSC1 (**c**) tumor cells after oHSV (MOI = 1, 24 h) treatment and IFNAR-1 blockade. All data are shown as the mean ± s.e.m. n = 3 independent experiments for **b**, **c**. Statistical significance was calculated via one-way ANOVA with Tukey's multiple comparisons test. n.s. not significant. Source data are provided as a Source Data file.

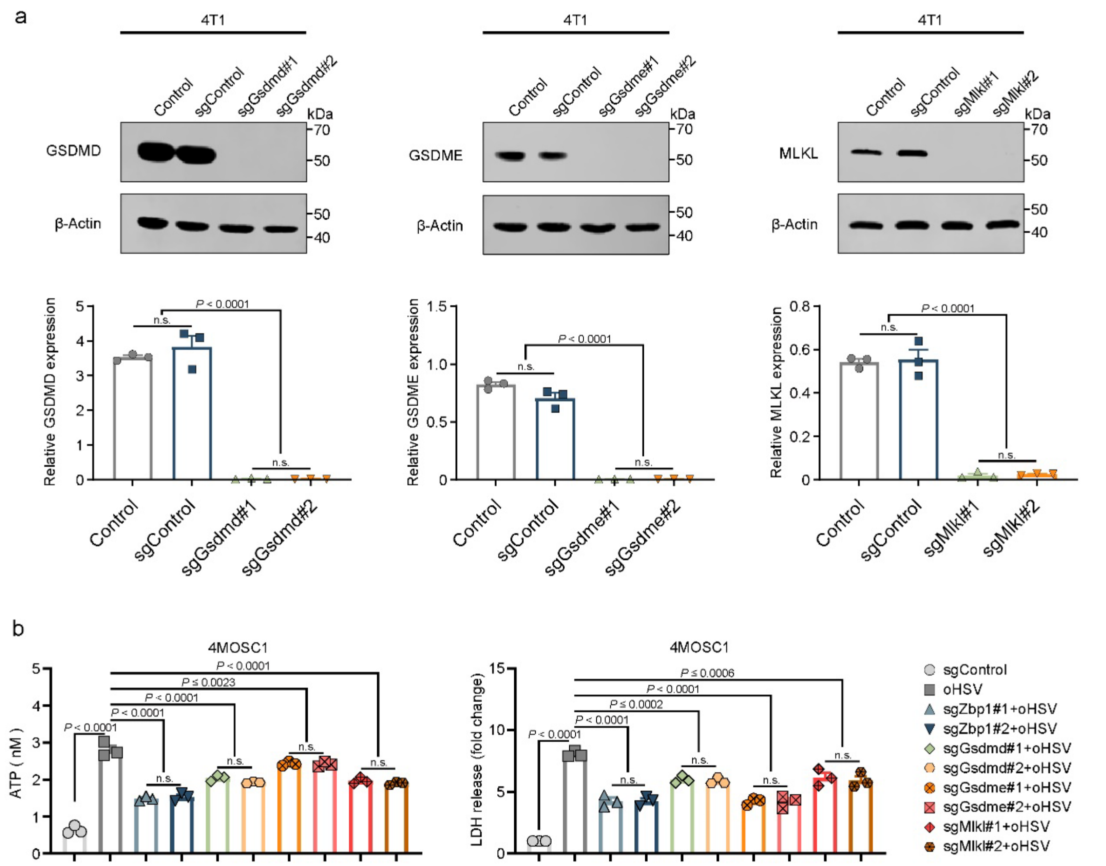

Supplementary Fig 3. Knockout verification of Gsdmd, Gsdme and Mlkl in 4T1 cells. a The situation of 4T1 cells transfected with sgRNA (*Gsdmd*, *Gsdme*, *Mlkl*) was detected by Western blotting. Western blotting was done thrice independently with similar results. **b** Detection of ATP and LDH release in 4MOSC1 cells from different treatment groups (n = 3 independent experiments). All data are shown as the mean ± s.e.m. Statistical significance was calculated via one-way ANOVA with Tukey's multiple comparisons test. n.s. not significant. Source data are provided as a Source Data file.

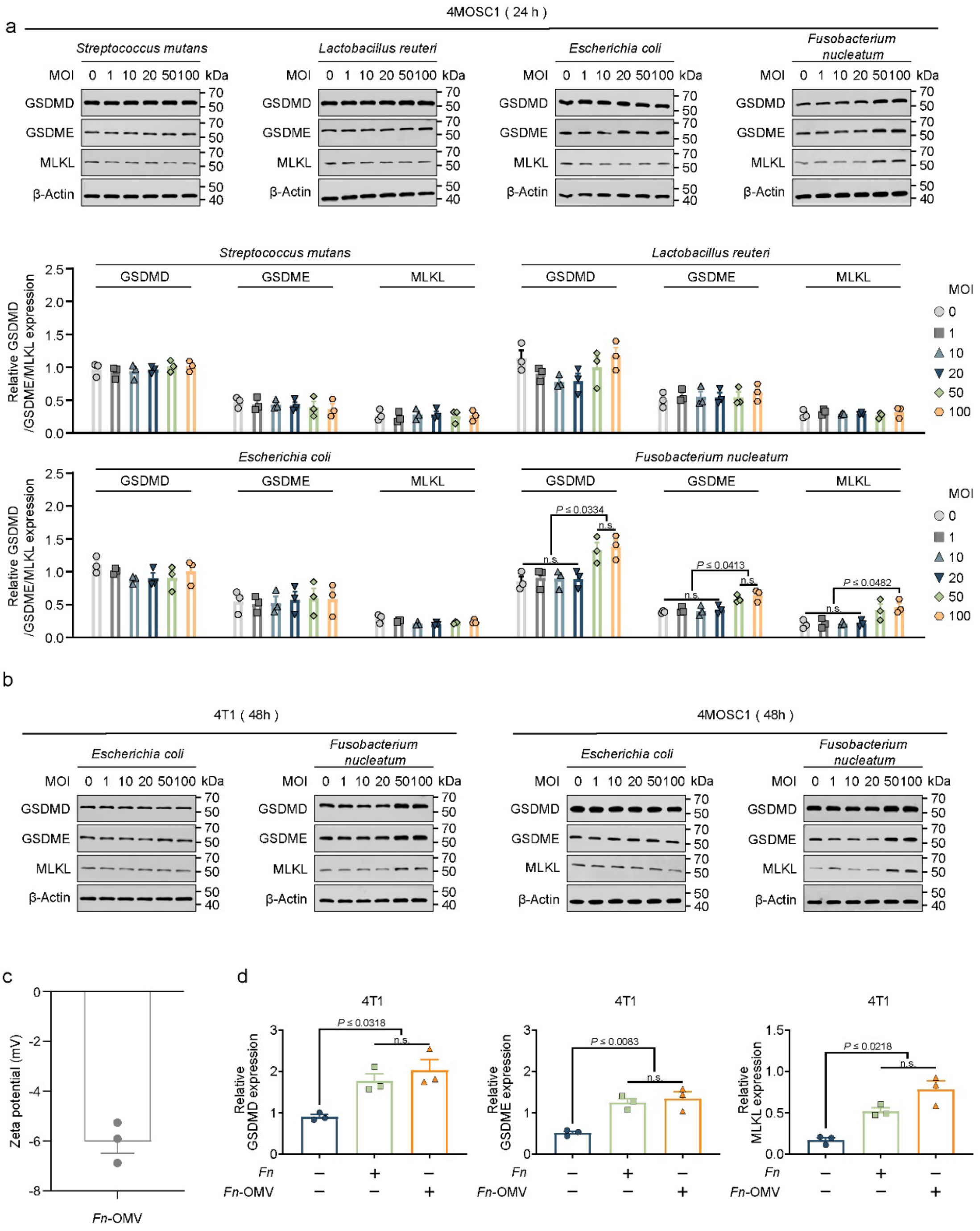

Supplementary Fig 4. *Fn*-OMV enhances the expression levels of PANoptosis effector proteins. a 4MOSC1 cells were infected with different bacterial titers (MOI = 0, 1, 10, 20, 50, 100) for 24 h, cellular protein was analyzed by Western blotting to detect the expression level of GSDMD, GSDME and MLKL (upper) and corresponding quantitative analyses (below). Western blotting was done thrice independently with similar results. **b** The protein expression of GSDMD, GSDME and MLKL was evaluated using western blotting in 4T1 (left) and 4MOSC1 (right) cells treated with *Escherichia coli* or *Fusobacterium nucleatum* of different MOI (MOI = 0, 1, 10, 20, 50, 100) for 24 h. Western blotting was done thrice independently with similar results. **c** Zeta potential of *Fn*-OMV (n = 3 independent experiments). **d** Cellular protein was

analyzed by Western blotting to detect corresponding quantitative analyses of GSDMD, GSDME, MLKL in 4T1 cells treated with *Fn* or *Fn*-OMV. Western blotting was done thrice independently with similar results. Statistical significance was calculated via one-way ANOVA with Tukey's multiple comparisons test. n.s. not significant. Source data are provided as a Source Data file.

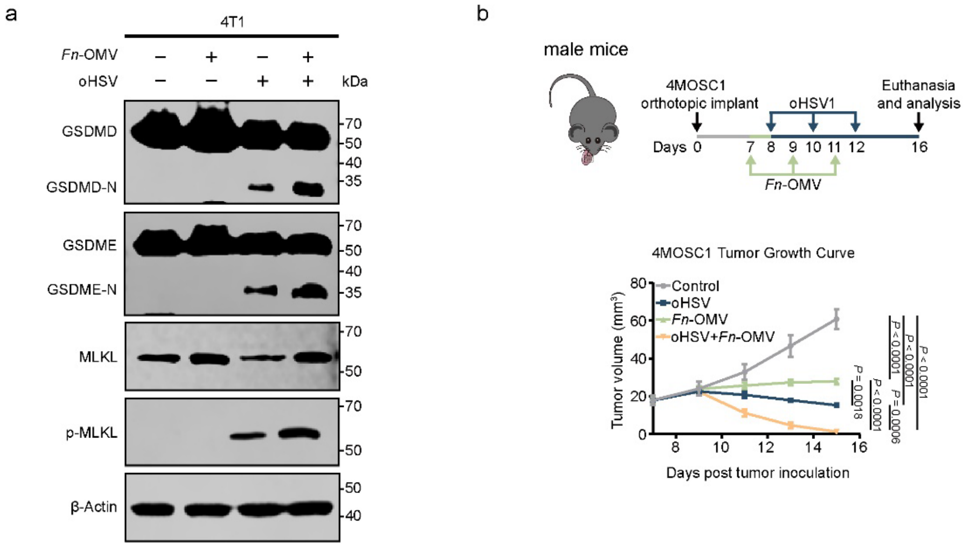

Supplementary Fig 5. The expression level of PANoptosis related genes. a Cellular protein was analyzed by Western blotting to detect the expression level of PANoptosis related genes in 4T1 cells treated with *Fn*-OMV (1 μg mL^{-1}) alone, oHSV (MOI = 1) alone or in combination. Western blotting was done thrice independently with similar results. **b** Schematic representation of 4MOSC1 tumor inoculation and treatment with oHSV and *Fn*-OMV. Average 4MOSC1 tumor growth curve depicted as mean ± s.e.m. for each treatment group (n = 5 mice). Statistical significance was evaluated using two-way ANOVA. Source data are provided as a Source Data file.

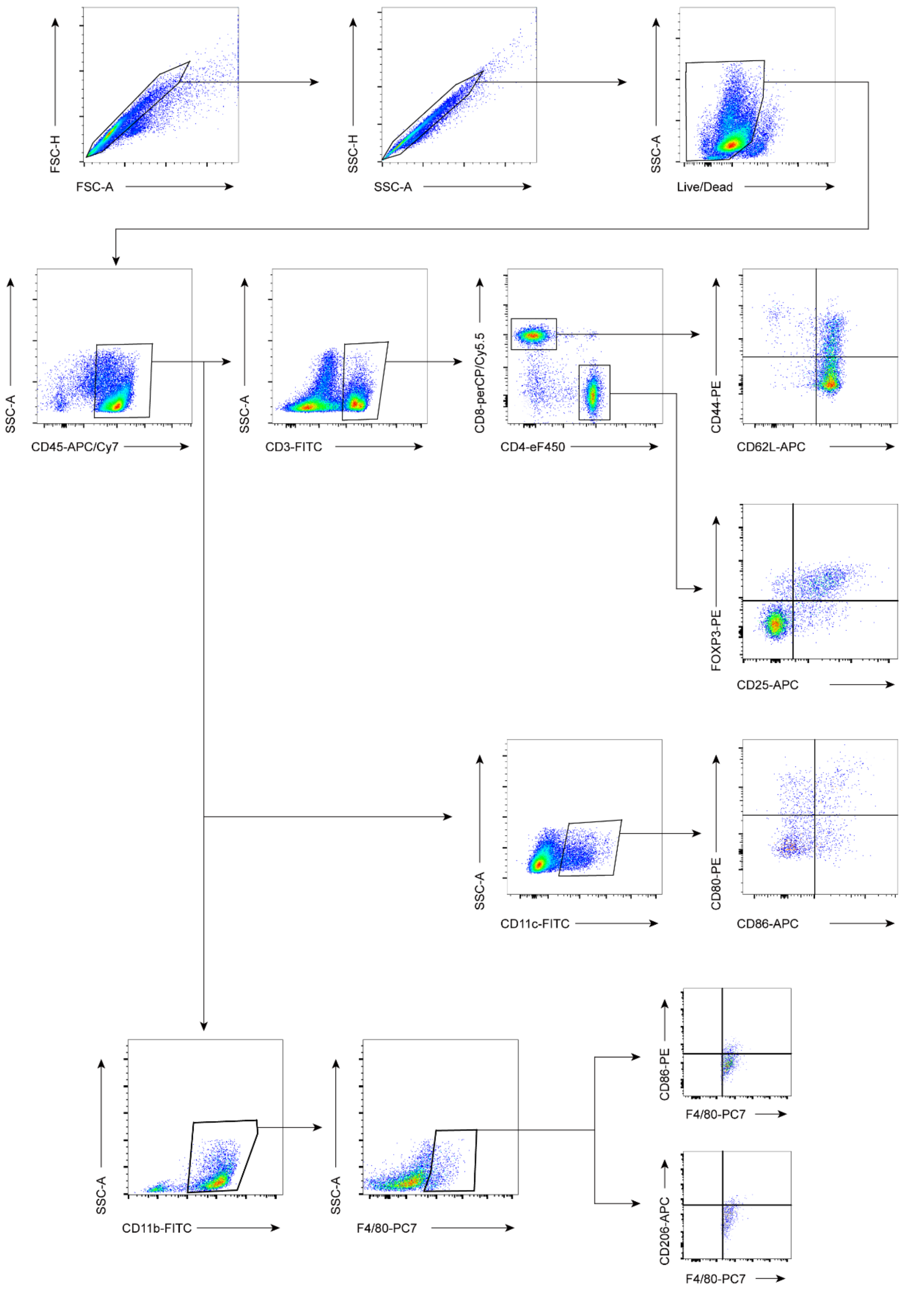

Supplementary Fig 6. Gating strategy. Gating strategy used to identify the CD45^{+} cells from tumor tissue and tumor draining lymph node (TDLN).

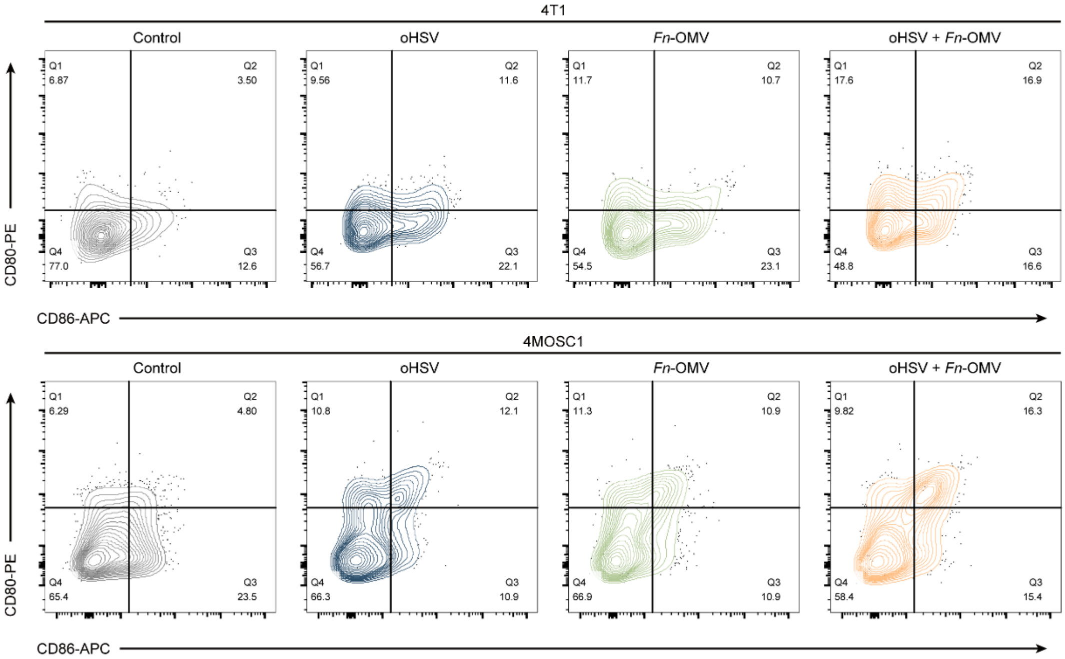

Supplementary Fig 7. Representative flow cytometric analysis images. Representative flow cytometric analysis images of $CD80^{+}$ and $CD86^{+}$ dendritic cell populations in the TDLNs (n = 5 independent experiments).

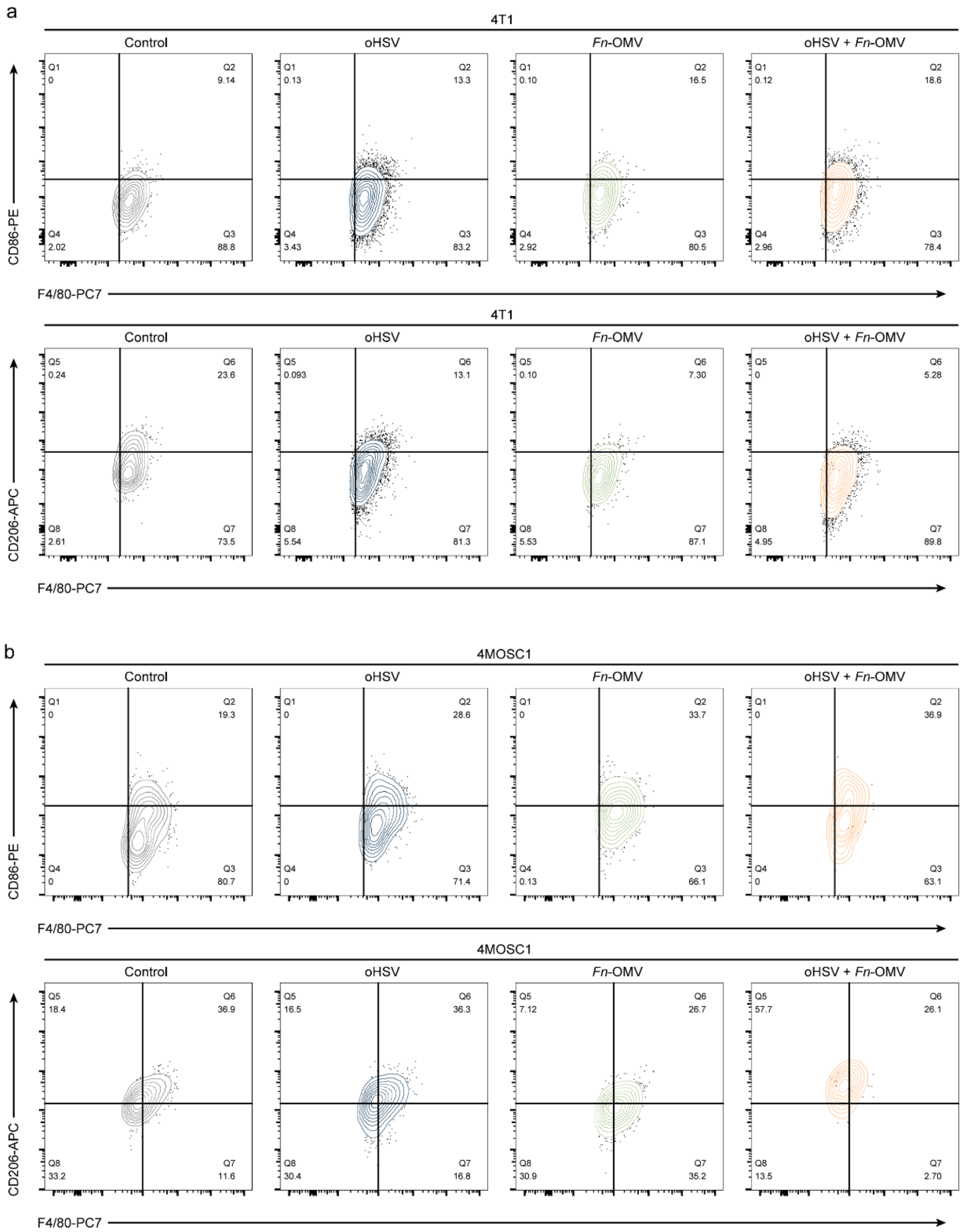

Supplementary Fig 8. Combination of Fn-OMV and oHSV converts "cold" tumors into "hot" tumors. a Representative flow cytometric analysis images of CD86$^+$ macrophages and CD206$^+$ macrophages in the tumors (4T1). **b** Representative flow cytometric analysis images of CD86$^+$ macrophages and CD206$^+$ macrophages in the tumors (4MOSC1). n = 5 independent experiments.

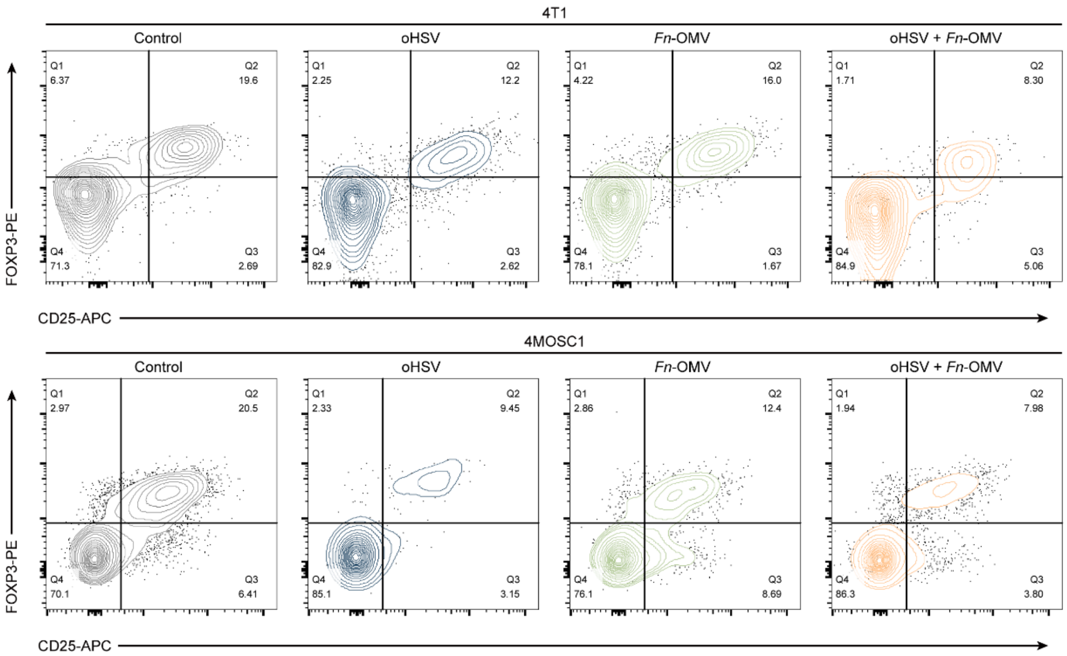

Supplementary Fig 9. Representative flow cytometric analysis images. Representative flow cytometric analysis images of $CD25^+$ and $FOXP3^+$ Treg cell populations in the TDLNs (n = 5 independent experiments).

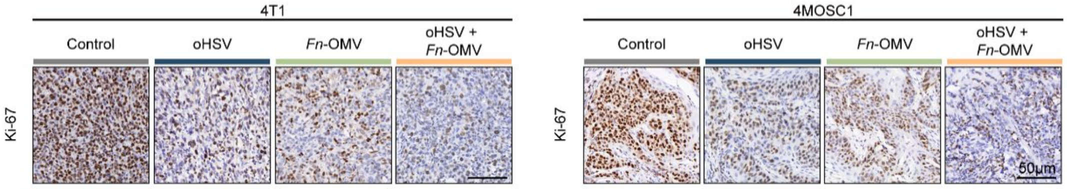

Supplementary Fig 10. Representative images of Ki-67 expression. Representative images of Ki-67 expression in different treatment groups of 4T1 tumors (left) and 4MOSC1 tumors (right), Scale bars, 50 μm. The images of immunostaining were representative of those generated from five mice each group with similar results.

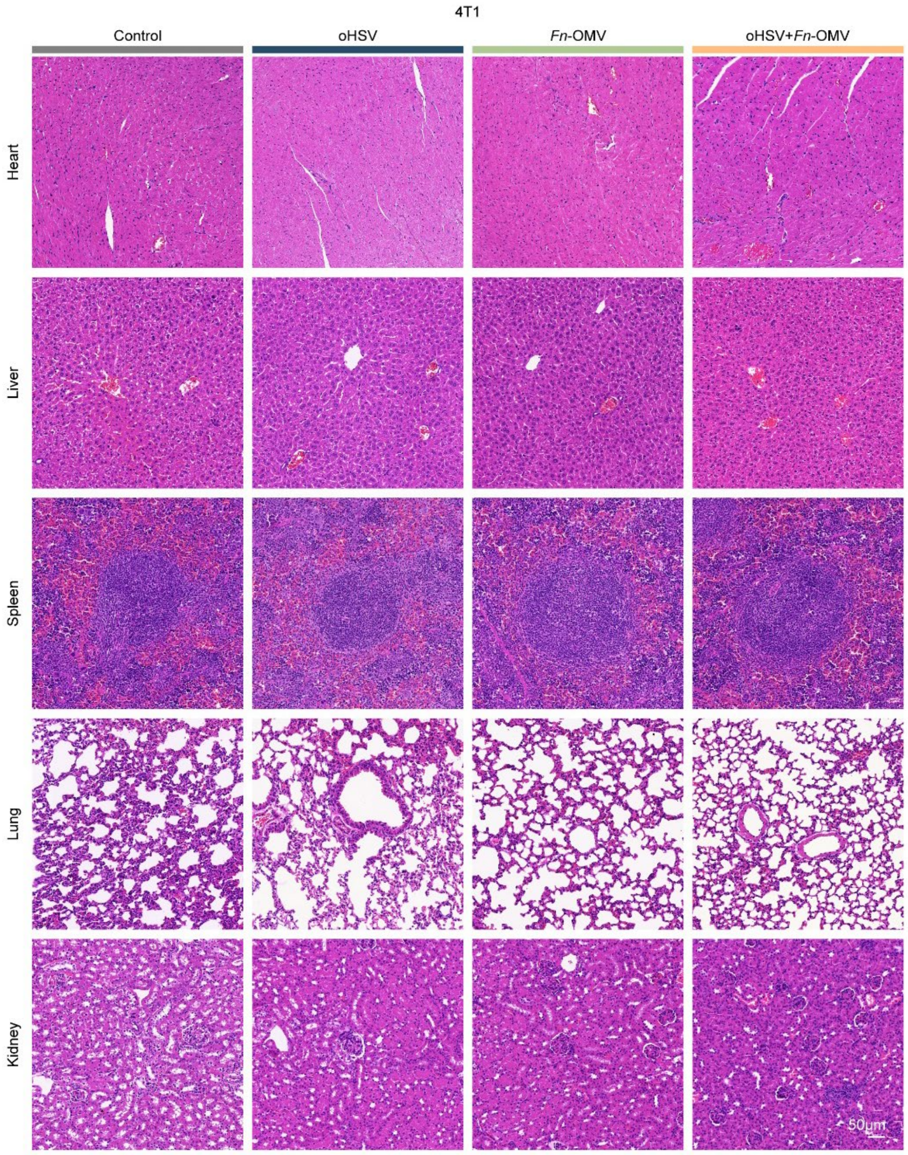

Supplementary Fig 11. Hematoxylin and eosin staining (H&E staining) results of heart, liver, spleen, lungs, and kidneys. H&E staining results of heart, liver, spleen, lungs, and kidneys in treated mice showed no abnormality in cellar morphology compared with control mice (BALB/c). Scale bars = 50 μm. The images of HE staining were representative of those generated from five mice each group with similar results.

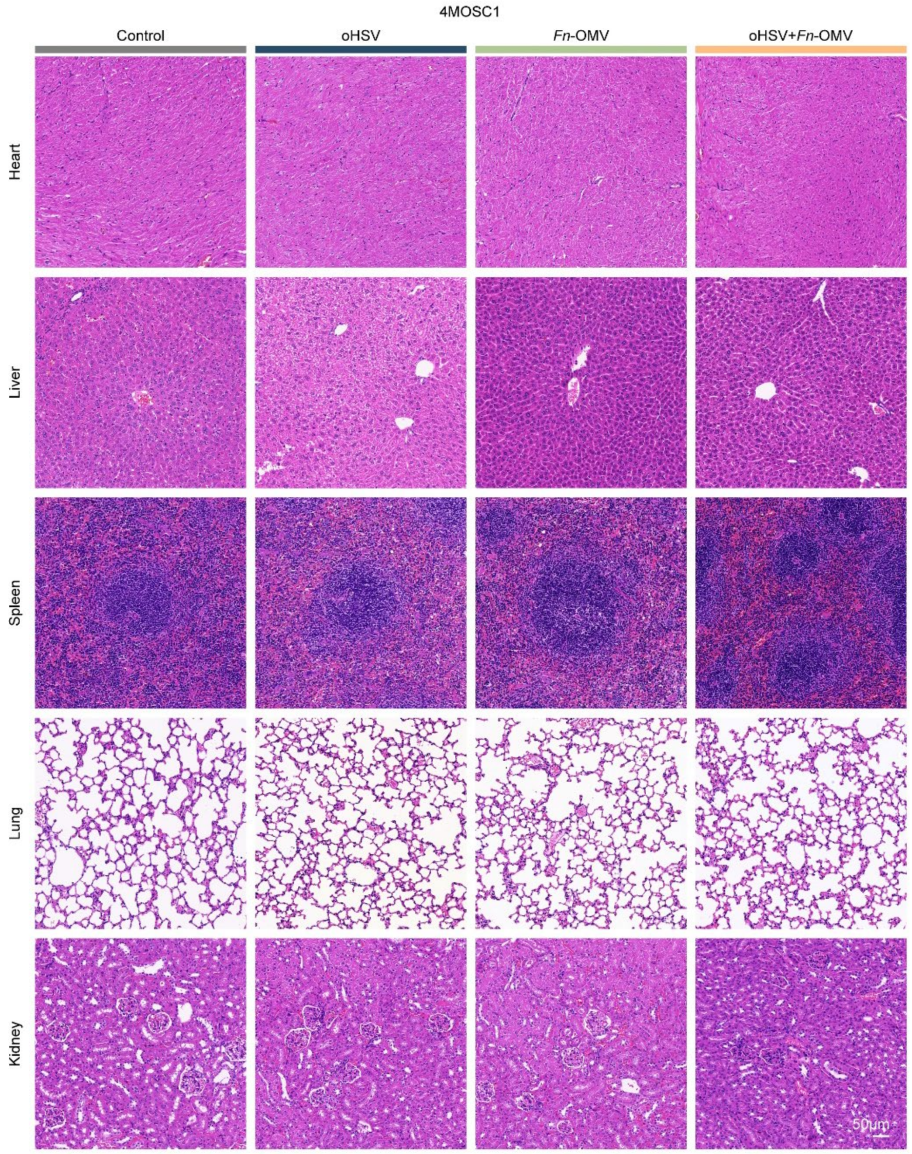

Supplementary Fig 12. Hematoxylin and eosin staining (H&E staining) results of heart, liver, spleen, lungs, and kidneys. H&E staining results of heart, liver, spleen, lungs, and kidneys in treated mice showed no abnormality in cellar morphology compared with control mice (C57Bl/6). Scale bars = 50 μm. The images of HE staining were representative of those generated from five mice each group with similar results.

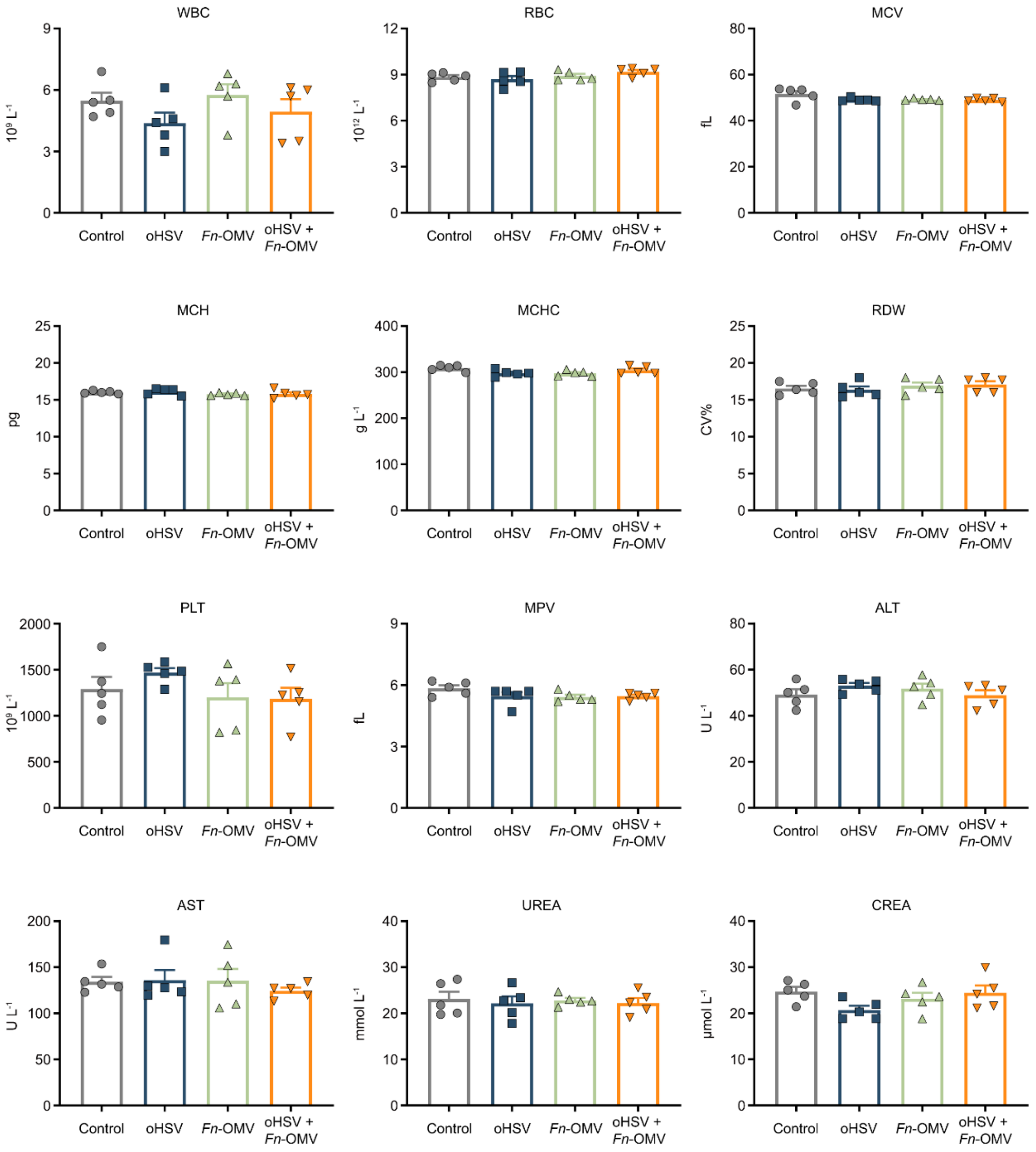

Supplementary Fig 13. Routine blood test and blood biochemical test of mice in the control and treated groups. WBC: white blood cell; RBC: red blood cell; MCV: mean corpuscular volume, MCH: mean corpuscular hemoglobin; MCHC: mean corpuscular hemoglobin concentration; RDW: red cell distribution width; PLT: platelets; MPV: mean platelet volume; ALT: Alanine aminotransferase; AST: Aspartate aminotransferase; UREA: carbamide; CREA: Creatinine. All data are shown as the mean ± s.e.m. n = 5 independent experiments. Statistical significance was calculated via one-way ANOVA with Tukey's multiple comparisons test. Source data are provided as a Source Data file.

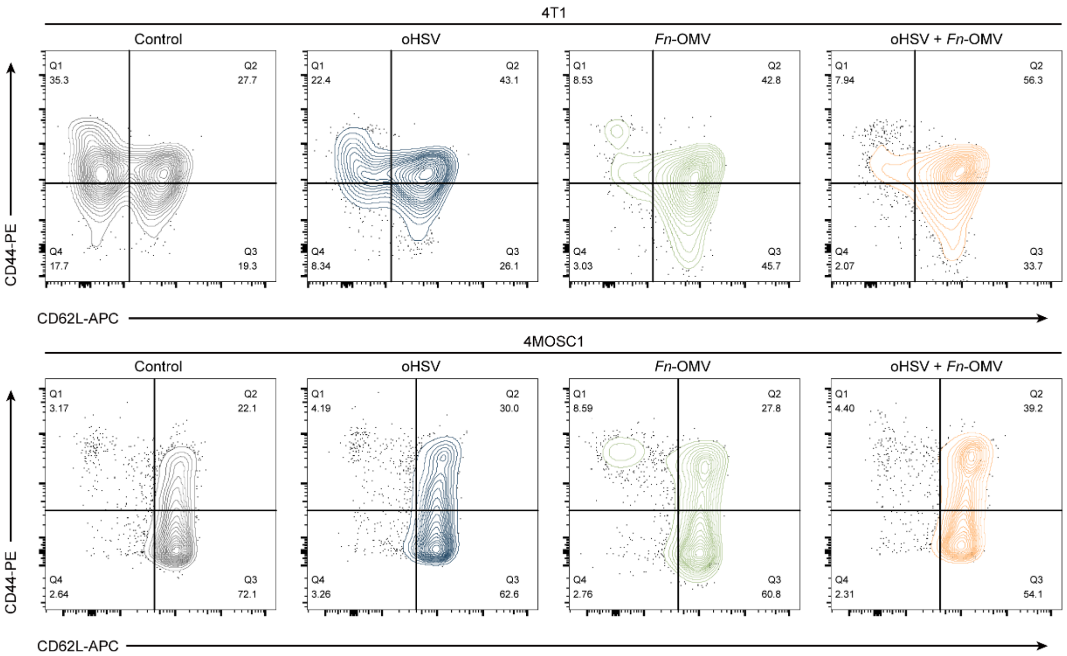

Supplementary Fig 14. Representative flow cytometric analysis images. Representative flow cytometric analysis images of $CD44^+$ and $CD62L^+$ T cell populations in the TDLNs (n = 5 independent experiments).

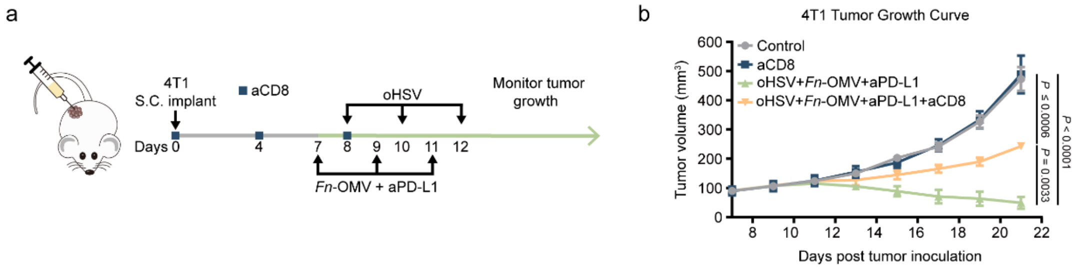

Supplementary Fig 15. $CD8^+$ T cells mediate tumor rejection responses. a Schematic representation of the immunization regimen for the killing assay and the timeline of animal experiments. S.C. = Subcutaneous. **b** Average tumor growth curve depicted as mean ± s.e.m. for each treatment group (n = 5 mice). Statistical significance was evaluated using two-way ANOVA. Source data are provided as a Source Data file.

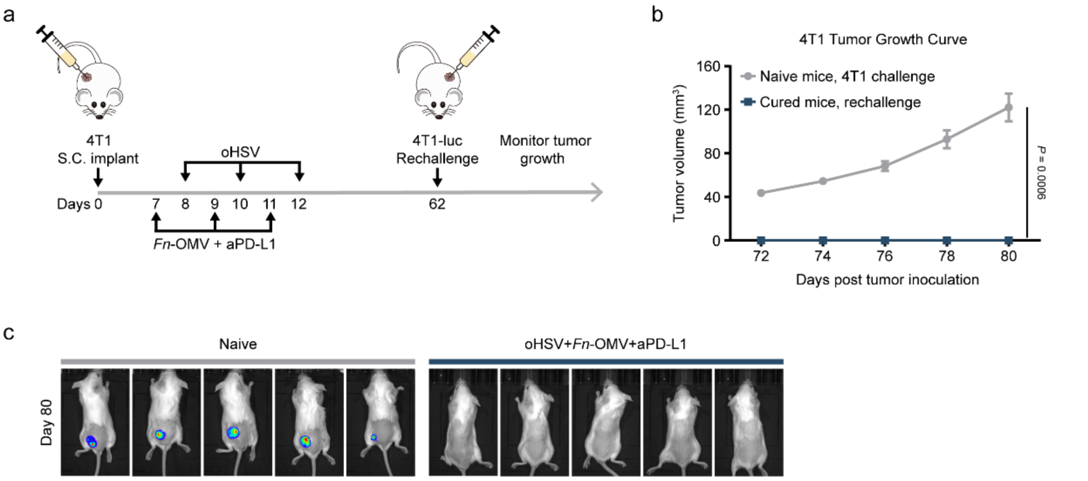

Supplementary Fig 16. Tumor challenge experiments in cured mice and naive mice. a Establishment of the rechallenge model. Tumor volume curves (**b**) and bioluminescence images (**c**) of naïve and oHSV + *Fn*-OMV + αPD-L1 treatment groups at different times; data are presented as mean ± s.e.m. (n = 5 mice). S.C. = Subcutaneous. Statistical significance was calculated via two-way ANOVA with Tukey's multiple comparisons test. Source data are provided as a Source Data file.

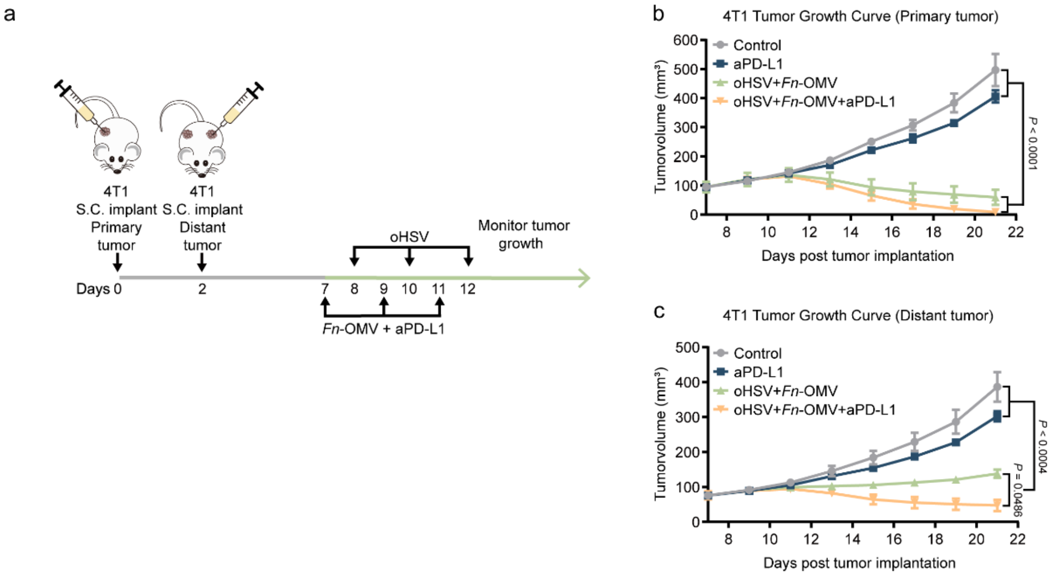

Supplementary Fig 17. A second flank tumor was rejected following injection of the primary flank tumor to demonstrate abscopal activity. a Schedule of oHSV + *Fn*-OMV + αPD-L1 combination therapy. The growth curves of primary (**b**) and distant tumor (**c**) of 4T1 tumor-bearing mice with different treatments. Data are presented as mean ± s.e.m. (n = 5 mice). S.C. = Subcutaneous. Statistical significance was calculated via two-way ANOVA with Tukey's multiple comparisons test. Source data are provided as a Source Data file.

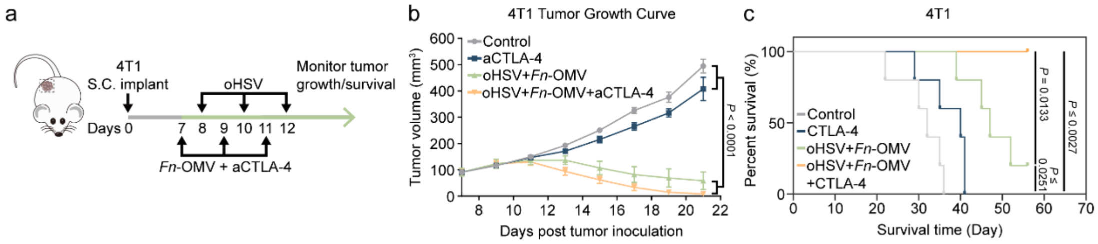

Supplementary Fig 18. Combination of *Fn*-OMV and oHSV enhances the effectiveness of anti-CTLA-4 immunotherapy. a Schedule of oHSV + *Fn*-OMV + αCTLA-4 combination therapy. S.C. = Subcutaneous. **b** Tumor volume of 4T1 tumor-bearing mice with different treatments (n = 5 mice). Statistical significance was calculated via two-way ANOVA with Tukey's multiple comparisons test. The growth curves of percent survival (**c**) of 4T1 tumor-bearing mice with different treatments. Data are presented as mean ± s.e.m. (n = 5 mice). Statistical significance was calculated via two-way ANOVA with Tukey's multiple comparisons test. Source data are provided as a Source Data file.

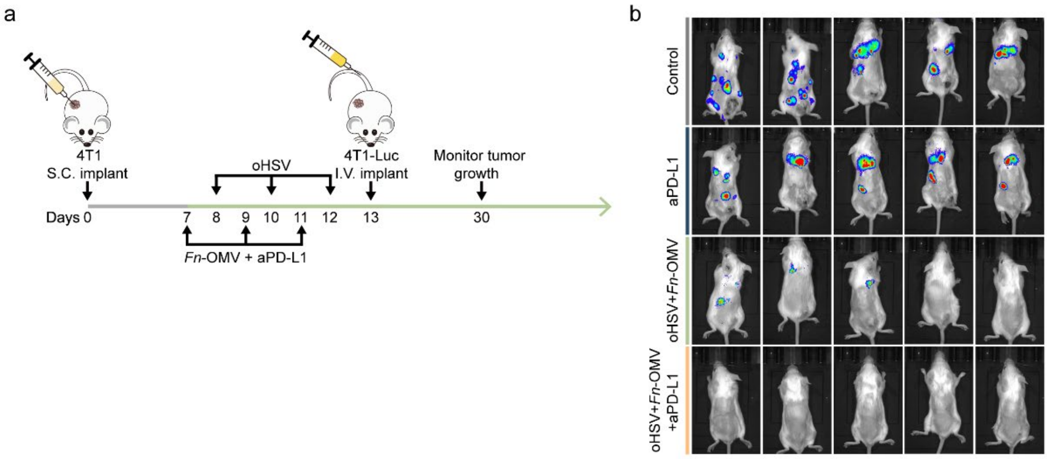

Supplementary Fig 19. Lung metastasis experiment. a Schematic showing the experiment treat mice with the 4T1 experimental model of metastasis at an early stage. BALB/c mice (n = 5 mice) were implanted subcutaneously with 1×10^6 4T1 mammary carcinoma cells on right hind flanks and after 13 days consequently implanted intravenously with 5×10^5 4T1-Luc cells to evaluated the effect of different treatment strategies on tumor metastasis in a lung metastasis mouse model. When primary tumor volumes were 100mm^3, mice received different treatments. S.C. = Subcutaneous. I.V.= intravenous. **b** Bioluminescence images tracking the spreading and growth of intravenously injected 4T1-Luc cells in BALB/c mice (n = 5 mice) treated with different treatment strategies on days 30.
