# Supplementary material for: Fn-OMV potentiates ZBP1-mediated PANoptosis triggered by oncolytic HSV-1 to fuel antitumor immunity
Source: Nat Commun. 2024 Apr 30;15:3669. doi: 10.1038/s41467-024-48032-7 (PMC11063137; doi:10.1038/s41467-024-48032-7)
Supplement: Supplementary file 3 — Description of Additional Supplementary Files [file 41467_2024_48032_MOESM3_ESM.pdf]

<u>Description of Additional Supplementary Files</u>

Supplementary Data Legend

Supplementary Data 1: Primer List
