# Supplementary material for: Fn-OMV potentiates ZBP1-mediated PANoptosis triggered by oncolytic HSV-1 to fuel antitumor immunity
Source: Nat Commun. 2024 Apr 30;15:3669. doi: 10.1038/s41467-024-48032-7 (PMC11063137; doi:10.1038/s41467-024-48032-7)
Supplement: Supplementary file 5 — Reporting Summary [file 41467_2024_48032_MOESM5_ESM.pdf]

nature portfolio

Corresponding author(s): Junjie Zhang, Zhi-Jun Sun

Last updated by author(s): Mar 29, 2024

Reporting Summary

Nature Portfolio wishes to improve the reproducibility of the work that we publish. This form provides structure for consistency and transparency in reporting. For further information on Nature Portfolio policies, see our Editorial Policies and the Editorial Policy Checklist.

Statistics

For all statistical analyses, confirm that the following items are present in the figure legend, table legend, main text, or Methods section.

n/a	Confirmed	
☐	☒	The exact sample size (n) for each experimental group/condition, given as a discrete number and unit of measurement
☐	☒	A statement on whether measurements were taken from distinct samples or whether the same sample was measured repeatedly
☐	☒	The statistical test(s) used AND whether they are one- or two-sided<br>*Only common tests should be described solely by name; describe more complex techniques in the Methods section.*
☐	☒	A description of all covariates tested
☐	☒	A description of any assumptions or corrections, such as tests of normality and adjustment for multiple comparisons
☐	☒	A full description of the statistical parameters including central tendency (e.g. means) or other basic estimates (e.g. regression coefficient) AND variation (e.g. standard deviation) or associated estimates of uncertainty (e.g. confidence intervals)
☐	☒	For null hypothesis testing, the test statistic (e.g. F, t, r) with confidence intervals, effect sizes, degrees of freedom and P value noted<br>*Give P values as exact values whenever suitable.*
☒	☐	For Bayesian analysis, information on the choice of priors and Markov chain Monte Carlo settings
☒	☐	For hierarchical and complex designs, identification of the appropriate level for tests and full reporting of outcomes
☒	☐	Estimates of effect sizes (e.g. Cohen's d, Pearson's r), indicating how they were calculated

Our web collection on statistics for biologists contains articles on many of the points above.

Software and code

Policy information about availability of computer code

Data collection	CytExpert software v. 2.3(Beckman), Image Studio software v. 5.2(Li-Cor Biosciences), Living Image software v.4.5 (PerkinElmer),ImageScope software v. 12.3.2 (Leica), ABI QuantStudio Software v1.3 (Thermo Fisher Scientific), FV10-ASW v.4.0 software (Olympus).
Data analysis	Softwares used for analysis include GraphPad Prism v. 8.0 (GraphPad Software), FlowJo v. 10 (TreeStar), Aperio ImageScope v. 12.3.2 (Leica), Image-Pro Plus v. 6.0 (Media Cybernetics), Excel 2016 (Microsoft), Living image software (PerkinElmer),Image Studio software v. 5.2 (Li-Cor Biosciences), ImageJ v. 1.8 (NationalInstitutes of Health), CaseViewer v. 2.4 (3DHISTECH).

For manuscripts utilizing custom algorithms or software that are central to the research but not yet described in published literature, software must be made available to editors and reviewers. We strongly encourage code deposition in a community repository (e.g. GitHub). See the Nature Portfolio guidelines for submitting code & software for further information.

Data

Policy information about availability of data

All manuscripts must include a data availability statement. This statement should provide the following information, where applicable:

- Accession codes, unique identifiers, or web links for publicly available datasets
- A description of any restrictions on data availability
- For clinical datasets or third party data, please ensure that the statement adheres to our policy

The data generated in this study are available within the Article, Supplementary Information or Source Data file. Source data are provided with this paper.

Research involving human participants, their data, or biological material

Policy information about studies with human participants or human data. See also policy information about sex, gender (identity/presentation), and sexual orientation and race, ethnicity and racism.

Reporting on sex and gender	N/A
Reporting on race, ethnicity, or other socially relevant groupings	N/A
Population characteristics	N/A
Recruitment	N/A
Ethics oversight	N/A

Note that full information on the approval of the study protocol must also be provided in the manuscript.

Field-specific reporting

Please select the one below that is the best fit for your research. If you are not sure, read the appropriate sections before making your selection.

☒ Life sciences ☐ Behavioural & social sciences ☐ Ecological, evolutionary & environmental sciences

For a reference copy of the document with all sections, see nature.com/documents/nr-reporting-summary-flat.pdf

Life sciences study design

All studies must disclose on these points even when the disclosure is negative.

Sample size	Although no sample size calculation was performed, sample sizes for the in vivo experiments are similar to those generally employed and accepted in the field (Nat Nanotechnol 14(1):89-97 (2019); Nat Biotechnol 37(11):1322-1331 (2019) and were sufficient to support our conclusions with statistical significance. Sample sizes for the in vitro experiments are also based on previous work (Nat Commun 4;12(1):6371 (2021)).
Data exclusions	No data were excluded.
Replication	Experiments were repeated at least twice to ensure reproducibility as indicated in the Figure legends. All attempts were successful. Exact numbers of biologically independent repetitions are stated in the manuscript.
Randomization	We excluded the mice that failed the model establishment. The experimental mice were grouped by random numbers table. During experiments, mice with the same sex were housed in the same cage. For experiments other than those involving mice, samples were also grouped by random numbers table.
Blinding	Investigators were not blinded during carrying out the experiment, but blinded during the allocation, sample collection, and data analysis. In vivo experiments were performed unblinded due to the requirements and regulations of the Institutional Animal Care and Use Committee (IACUC) guidelines of Wuhan University.

Reporting for specific materials, systems and methods

We require information from authors about some types of materials, experimental systems and methods used in many studies. Here, indicate whether each material, system or method listed is relevant to your study. If you are not sure if a list item applies to your research, read the appropriate section before selecting a response.

Materials & experimental systems

n/a	Involved in the study	
☐	☒	Antibodies
☐	☒	Eukaryotic cell lines
☒	☐	Palaeontology and archaeology
☐	☒	Animals and other organisms
☒	☐	Clinical data
☒	☐	Dual use research of concern
☒	☐	Plants

Methods

n/a	Involved in the study	
☒	☐	ChIP-seq
☐	☒	Flow cytometry
☒	☐	MRI-based neuroimaging

Antibodies

Antibodies used

The following primary antibodies were used for blocking. They are listed as antigen first, followed by supplier and clone/catalog number as applicable.
1) anti-mouse PD-L1 (aPD-L1), 200 μg per mouse, Bioxcell, Clone 10F.9G2™, Cat BE0101.
2) anti-mouse IFNAR-1 (aIFNAR-1), 100 ng mL−1, Bioxcell, Clone MAR1-5A3, Cat BP0241.
3) anti-mouse CD8 (aCD8), 200 μg per mouse, Bioxcell, Clone 53-6.7, Cat BE0004-1.
4) anti-mouse CTLA-4 (aCTLA-4), 200 μg per mouse, Bioxcell, Clone 9D9, Cat BE0164.
The following antibodies were used for western blotting. They are listed as antigen first, followed by supplier, catalog number as applicable.
1)anti-GSDMD, 1:1000, Abcam, Cat ab219800;
2)anti-GSDME, 1:1000, Abcam, Cat ab215191;
3)anti-MLKL, 1:1000, Cell Signaling Technology, Cat 37705;
4)anti-Phospho-MLKL, 1:1000, Cell Signaling Technology, Cat 37333;
5)anti-beta Actin, 1:1000, Abcam, Cat ab8226;
6)anti-Caspase-3, 1:1000, Cell Signaling Technology, Cat 9662;
7)anti-Cleaved Caspase-3, 1:1000, Cell Signaling Technology, Cat 9661;
8)anti-ZBP1, 1:1000, AdipoGen, Cat AG-20B-0010;
9)anti-NLRP6, 1:1000, ABclonal, Cat A15628;
10)anti-Ubiquitin, 1:1000, Proteintech, Cat 10201;
11)HRP-conjugated Affinipure Goat Anti-Mouse IgG(H+L), 1:5000,Proteintech, Cat SA00001-1;
12)HRP-conjugated Affinipure Goat Anti-Rabbit IgG(H+L), 1:5000,Proteintech, Cat SA00001-2;
13)anti-Lamin A/C, 1:2000, Cell Signaling Technology, Cat 4777;
The following antibodies were used for immunohistochemistry (IHC), Multiplexed Immunohistochemistry, and immunofluorescence. They are listed as antigen first, followed by supplier, catalog number as applicable.
1)anti-Ki67, 1:400, Abcam, Cat ab15580;
2)anti-CD8α, 1:400, Cell Signaling Technology, Cat 98941;
3)anti-Foxp3, 1:400, Cell Signaling Technology, Cat 12653;
4)anti-granzyme B, 1:200, Cell Signaling Technology, Cat 44153;
5)anti-HMGB1 1:100 , Abcam, Cat ab18256;
6)anti-ZBP1, 1:400, AdipoGen, Cat AG-20B-0010;
7)anti-Z-DNA, 1:400, Absolute Antibody, Cat Ab00783-3.0;
8)Goat anti-Rabbit IgG DyLight 488 , 1:200, abbkine, Cat A23220;
9)PD-L1, 1:200, Cell Signaling Technology, Cat 64988;
10)F4/80, 1:200, Cell Signaling Technology, Cat 70076;
The following primary antibodies were used for flow cytometry. They are listed as antigen first, followed by supplier and clone/catalog number as applicable.
1)Fixable Viability Dye, 1:1000, eBioscience, eFluor 506, Cat 65-0866-14
2)anti-mouse CD45, 1:500, eBioscience, APC-Cyanine7, Clone I3/2.3, Cat A15395;
3)anti-mouse CD3e, 1:500, eBioscience, FITC, Clone 145-2C11, Cat 11-0031-82;
4)anti-mouse CD4, 1:500, eBioscience, eFluor 450, Clone RM4-5, Cat 48-0042-82;
5)anti-mouse CD8a, 1:500, Biolegend, PerCP, Clone 5H10, Cat MCD0831;
6)anti-mouse CD11b, 1:500, Biolegend, FITC, Clone M1/70, Cat 101206;
7)anti-mouse CD11c, 1:500, eBioscience, FITC, Clone N418, Cat 11-0114-82;
8)anti-mouse CD80, 1:300, eBioscience, PE, Clone 16-10A1, Cat 12-0801-82;
9)anti-mouse CD86, 1:300, eBioscience, APC, Clone GL1, Cat 17-0862-82;
10)anti-mouse MHC-II, 1:300, eBioscience, PE-Cyanine7, Clone M5/114.15.2, Cat 5321-82;
11)anti-mouse CD25, 1:200, eBioscience, APC, Clone PC61.5, Cat 17-0251-82;
12)anti-mouse Foxp3, 1:100, eBioscience, PE, Clone FJK-16s, Cat 12-5773-82;
13)anti-mouse CD44, 1:500, eBioscience, PE, Clone IM7, Cat 12-0441-82;
14)anti-mouse CD62L, 1:200, eBioscience, APC, Clone MEL-14, Cat 17-0621-82;
15)anti-mouse F4/80, 1:200, eBioscience, PE-Cyanine7, Clone BM8, Cat 25-4801-82
16)anti-mouse CD206, 1:200, Biolegend, PE, Clone C068C2, Cat 141705
17)anti-mouse CD86, 1:200, Biolegend, PE, Clone A17199A, Cat 159204;
18)anti-mouse PD-L1, 1:200, Biolegend, PE, Clone 10F.9G2, Cat 124308;

Validation

No customized antibodies were used. Validation data of the antibodies purchased from commercial vendors are available on the manufactures' website and datasheets.
1) anti-mouse PD-L1 (aPD-L1), 200 μg per mouse, Bioxcell, Clone 10F.9G2™, Cat BE0101.
https://bioxcell.com/invivomab-anti-mouse-pd-l1-b7-h1-be0101
2) anti-mouse IFNAR-1 (aIFNAR-1), 100 ng mL−1, Bioxcell, Clone MAR1-5A3, Cat BP0241.
https://bioxcell.com/invivoplus-anti-mouse-ifnar-1
3) anti-GSDMD, 1:1000, Abcam, Cat ab219800;
https://www.abcam.cn/products/primary-antibodies/gsdmd-antibody-epr20859-ab219800.html
4) anti-GSDME, Abcam, Cat ab215191
https://www.abcam.cn/products/primary-antibodies/dfna5gsdme-antibody-epr19859-n-terminal-ab215191.html
5) anti-MLKL, 1:1000, Cell Signaling Technology, Cat 37705;
https://www.cellsignal.cn/products/primary-antibodies/mlkl-d6w1k-rabbit-mab/37705?site-searchtype=Products&N=4294956287&Ntt=37705&fromPage=plp&_requestid=1196099
6) anti-Phospho-MLKL, 1:1000, Cell Signaling Technology, Cat 37333;
https://www.cellsignal.cn/products/primary-antibodies/phospho-mlkl-ser345-d6e3g-rabbit-mab/37333?site-searchtype=Products&N=4294956287&Ntt=37333&fromPage=plp&_requestid=1196557

7) anti-beta Actin, 1:1000, Abcam, Cat ab8226;
https://www.abcam.cn/products/primary-antibodies/beta-actin-antibody-mabcam-8226-loading-control-ab8226.html
8) anti-Caspase-3, 1:1000, Cell Signaling Technology, Cat 9662;
https://www.cellsignal.cn/products/primary-antibodies/caspase-3-antibody/9662?site-search-type=Products&N=4294956287&Ntt=9662&fromPage=plp
9) anti-Cleaved Caspase-3, 1:1000, Cell Signaling Technology, Cat 9661;
https://www.cellsignal.cn/products/primary-antibodies/cleaved-caspase-3-asp175-antibody/9661?site-searchtype=Products&N=4294956287&Ntt=9661&fromPage=plp&_requestid=1197026
10) anti-ZBP1, 1:1000, AdipoGen, Cat AG-20B-0010;
https://adipogen.com/ag-20b-0010-anti-zbp1-mab-zippy-1.html
11) anti-NLRP6, 1:1000, ABclonal, Cat A15628;
https://abclonal.com.cn/catalog/A15628
12) anti-Ubiquitin, 1:1000, Proteintech, Cat 10201;
https://www.ptgcn.com/products/ubiquitin-Antibody-10201-2-AP.htm
13) HRP-conjugated Affinipure Goat Anti-Mouse IgG(H+L), 1:5000,Proteintech, Cat SA00001-1;
https://www.ptgcn.com/products/HRP-conjugated-Affinipure-Goat-Anti-Mouse-IgG-H-L-secondary-antibody.htm
14) HRP-conjugated Affinipure Goat Anti-Rabbit IgG(H+L), Proteintech, Cat SA00001-2
https://www.ptgcn.com/products/HRP-conjugated-Affinipure-Goat-Anti-Rabbit-IgG-H-L-secondary-antibody.htm
15) anti-Ki67, 1:400, Abcam, Cat ab15580;
https://www.abcam.cn/products/primary-antibodies/ki67-antibody-ab15580.html
16) anti-CD8α, 1:400, Cell Signaling Technology, Cat 98941;
https://www.cellsignal.com/products/primary-antibodies/cd8a-d4w2z-xp-rabbit-mab-mouse-specific/98941
17) anti-Foxp3, 1:400, Cell Signaling Technology, Cat 12653;
https://www.cellsignal.cn/products/primary-antibodies/foxp3-d6o8r-rabbit-mab/12653?site-searchtype=Products&N=4294956287&Ntt=12653&fromPage=plp&_requestid=1200826
18) anti-granzyme B, 1:200, Cell Signaling Technology, Cat 44153;
https://www.cellsignal.com/products/primary-antibodies/granzyme-b-e5v2l-rabbit-mab-mouse-specific/44153
19) anti-HMGB1 1:100 , Abcam, Cat ab18256;
https://www.abcam.cn/products/primary-antibodies/hmgb1-antibody-ab18256.html
20) anti-ZBP1, 1:400, AdipoGen, Cat AG-20B-0010;
https://adipogen.com/ag-20b-0010-anti-zbp1-mab-zippy-1.html
21) anti-Z-DNA, 1:400, Absolute Antibody, Cat Ab00783-3.0;
https://absoluteantibody.com/product/anti-z-dna-z22/Ab00783-3.0_mouse_igg2b/
22) Goat anti-Rabbit IgG DyLight 488 , 1:200, abbkine, Cat A23220; https://www.abbkine.cn/product/a23220/
23) PD-L1, 1:200, Cell Signaling Technology, Cat 64988;
https://www.cellsignal.cn/products/primary-antibodies/pd-l1-d5v3b-rabbit-mab/64988?site-searchtype=Products&N=4294956287&Ntt=64988&fromPage=plp&_requestid=1201388
24) F4/80, 1:200, Cell Signaling Technology, Cat 70076;
https://www.cellsignal.cn/products/primary-antibodies/f4-80-d2s9r-xp-rabbit-mab/70076?site-searchtype=Products&N=4294956287&Ntt=70076&fromPage=plp&_requestid=1201461
25) Fixable Viability Dye, 1:1000, eBioscience, eFluor 506, Cat 65-0866-14
https://www.thermofisher.cn/order/catalog/product/65-0866-14?SID=srch-srp-65-0866-14
26) anti-mouse CD45, 1:500, eBioscience, APC-Cyanine7, Clone I3/2.3, Cat A15395;
https://www.thermofisher.cn/cn/zh/antibody/product/CD45-Antibody-clone-30-F11-Monoclonal/45-0451-82
27) anti-mouse CD3e, 1:500, eBioscience, FITC, Clone 145-2C11, Cat 11-0031-82;
https://www.thermofisher.cn/cn/zh/antibody/product/CD3e-Antibody-clone-145-2C11-Monoclonal/11-0031-82
28) anti-mouse CD4, 1:500, eBioscience, eFluor 450, Clone RM4-5, Cat 48-0042-82;
https://www.thermofisher.cn/cn/zh/antibody/product/CD4-Antibody-clone-RM4-5-Monoclonal/48-0042-82
29) anti-mouse CD8a, 1:500, Biolegend, PerCP, Clone 5H10, Cat MCD0831;
https://www.thermofisher.cn/cn/zh/antibody/product/CD8-alpha-Antibody-clone-5H10-Monoclonal/MCD0831
30) anti-mouse CD11b, 1:500, Biolegend, FITC, Clone M1/70, Cat 101206;
https://www.biolegend.com/en-us/products/fitc-anti-mouse-human-cd11b-antibody-347
31) anti-mouse CD11c, 1:500, eBioscience, FITC, Clone N418, Cat 11-0114-82;
https://www.thermofisher.cn/cn/zh/antibody/product/CD11c-Antibody-clone-N418-Monoclonal/11-0114-82
32) anti-mouse CD80, 1:300, eBioscience, PE, Clone 16-10A1, Cat 12-0801-82;
https://www.thermofisher.cn/cn/zh/antibody/product/CD80-B7-1-Antibody-clone-16-10A1-Monoclonal/12-0801-82
33) anti-mouse CD86, 1:300, eBioscience, APC, Clone GL1, Cat 17-0862-82;
https://www.thermofisher.cn/cn/zh/antibody/product/CD86-B7-2-Antibody-clone-GL1-Monoclonal/17-0862-82
34) anti-mouse MHC-II, 1:300, eBioscience, PE-Cyanine7, Clone M5/114.15.2, Cat 5321-82;
https://www.thermofisher.cn/cn/zh/antibody/product/MHC-Class-II-I-A-I-E-Antibody-clone-M5-114-15-2-Monoclonal/25-5321-82
35)anti-mouse CD25, 1:200, eBioscience, APC, Clone PC61.5, Cat 17-0251-82;
https://www.thermofisher.cn/cn/zh/antibody/product/CD25-Antibody-clone-PC61-5-Monoclonal/17-0251-82
36)anti-mouse Foxp3, 1:100, eBioscience, PE, Clone FJK-16s, Cat 12-5773-82;
https://www.thermofisher.cn/cn/zh/antibody/product/FOXP3-Antibody-clone-FJK-16s-Monoclonal/12-5773-82
37)anti-mouse CD44, 1:500, eBioscience, PE, Clone IM7, Cat 12-0441-82;
https://www.thermofisher.cn/cn/zh/antibody/product/CD44-Antibody-clone-IM7-Monoclonal/12-0441-82
38)anti-mouse CD62L, 1:200, eBioscience, APC, Clone MEL-14, Cat 17-0621-82;
https://www.thermofisher.cn/cn/zh/antibody/product/CD62L-L-Selectin-Antibody-clone-MEL-14-Monoclonal/17-0621-82
39)anti-mouse F4/80, 1:200, eBioscience, PE-Cyanine7, Clone BM8, Cat 25-4801-82
https://www.thermofisher.cn/cn/zh/antibody/product/F4-80-Antibody-clone-BM8-Monoclonal/25-4801-82
40)anti-mouse CD206, 1:200, Biolegend, PE, Clone C068C2, Cat 141705
https://www.biolegend.com/en-us/products/pe-anti-mouse-cd206-mmr-antibody-7424
41)anti-mouse CD86, 1:200, Biolegend, PE, Clone A17199A, Cat 159204;
https://www.biolegend.com/en-us/products/pe-anti-mouse-cd86-antibody-18945
42)anti-mouse PD-L1, 1:200, Biolegend, PE, Clone 10F.9G2, Cat 124308;
https://www.biolegend.com/en-us/products/pe-anti-mouse-cd274-b7-h1-pd-l1-antibody-4497

43)anti-mouse CD8 (aCD8), 200 µg per mouse, Bioxcell, Clone 53-6.7, Cat BE0004-1.
https://bioxcell.com/invivomab-anti-mouse-cd8a-be0004-1
44)anti-mouse CTLA-4 (aCTLA-4), 200 µg per mouse, Bioxcell, Clone 9D9, Cat BE0164.
https://bioxcell.com/invivomab-anti-mouse-ctla-4-cd152-be0164
45)anti-Lamin A/C, 1:2000, Cell Signaling Technology, Cat 4777;
https://www.cellsignal.cn/products/primary-antibodies/lamin-a-c-4c11-mouse-mab/4777

Eukaryotic cell lines

Policy information about cell lines and Sex and Gender in Research

Cell line source(s)	The murine breast cancer cell line 4T1 (Cat CRL-2539), obtained from the American Type Culture Collection (ATCC). The murine colon carcinoma cell line CT26 (Cat TCM37), purchased from the Shanghai Cell Bank of the Chinese Academy of Sciences. RAW 264.7 (Cat TIB-71) obtained from the ATCC. The 4MOSC1 and 4MOSC2 cells were gifts from Prof. J. Silvio Gutkind at the University of California San Diego via a material transfer agreement (SD2017-202)(PMID:31804466). The SCC7 cells were gifts from Prof. Qianming Chen (West China School and Hospital of Stomatology Sichuan University) (PMID:25177028).
Authentication	Each cell line used was morphologically confirmed according to the information provided by culture collections.
Mycoplasma contamination	All the cell lines presented in this study were tested for mycoplasma contamination and they were free of mycoplasma contamination.
Commonly misidentified lines (See ICLAC register)	No commonly misidentified cell lines were used.

Animals and other research organisms

Policy information about studies involving animals; ARRIVE guidelines recommended for reporting animal research, and Sex and Gender in Research

Laboratory animals	Female C57BL/6, male C57BL/6 and female BALB/c mice, aged 6-8 weeks and weighing 18-20g, were obtained from Hubei Provincial Academy of Preventive Medicine.
Wild animals	The study did not involve wild animals.
Reporting on sex	This study did not involve sex.
Field-collected samples	The study did not involve samples collected from field.
Ethics oversight	Ethical approval for this study was granted by the Animal Ethics Committee of the School and Hospital of Stomatology of Wuhan University (approval number: S07922090D). All animal experimental procedures were performed in accordance with the Regulations for the Administration of Affairs Concerning Experimental Animals approved by the State Council of the People's Republic of China.

Note that full information on the approval of the study protocol must also be provided in the manuscript.

Plants

Seed stocks	N/A
Novel plant genotypes	N/A
Authentication	N/A

Flow Cytometry

Plots

Confirm that:

☒ The axis labels state the marker and fluorochrome used (e.g. CD4-FITC).

☒ The axis scales are clearly visible. Include numbers along axes only for bottom left plot of group (a 'group' is an analysis of identical markers).

☒ All plots are contour plots with outliers or pseudocolor plots.

☒ A numerical value for number of cells or percentage (with statistics) is provided.

Methodology

Sample preparation	For lymphocyte analysis, tumors and tumor-draining lymph nodes were collected from mice and made into cell suspensions by gentleMACS™ dissociator and digestive enzyme (Miltenyi Biotec) according to the manufacturer's instructions. Then, the samples were passed through 200-mesh nylon mesh filters to obtain single-cell suspensions. Live or dead cells were separated by Fixable Viability Dye (eBioscience, eFluor 780). For all samples, cells were first stained with antibodies against surface antigens. In some experiments, cells were subsequently fixed, permeablized and stained for intracellular antigens.
Instrument	CytoFLEX flow cytometer (Beckman), MoFlo XDP cell sorter (Beckman)
Software	CytExpert v. 2.3 software (Beckman), FlowJo v. 10 (TreeStar)
Cell population abundance	The purity of the post-sorted cells was more than 95% as verified by flow cytometry.
Gating strategy	The gating strategy was shown in Supplementary Fig. 4. Generally, single cell gates based on SSC-H and SSC-A, and FSC-H and FSC-A were used to exclude non-singlets. A live/dead cell gate based on fixable viability dye was used to exclude dead cells.

☒ Tick this box to confirm that a figure exemplifying the gating strategy is provided in the Supplementary Information.
